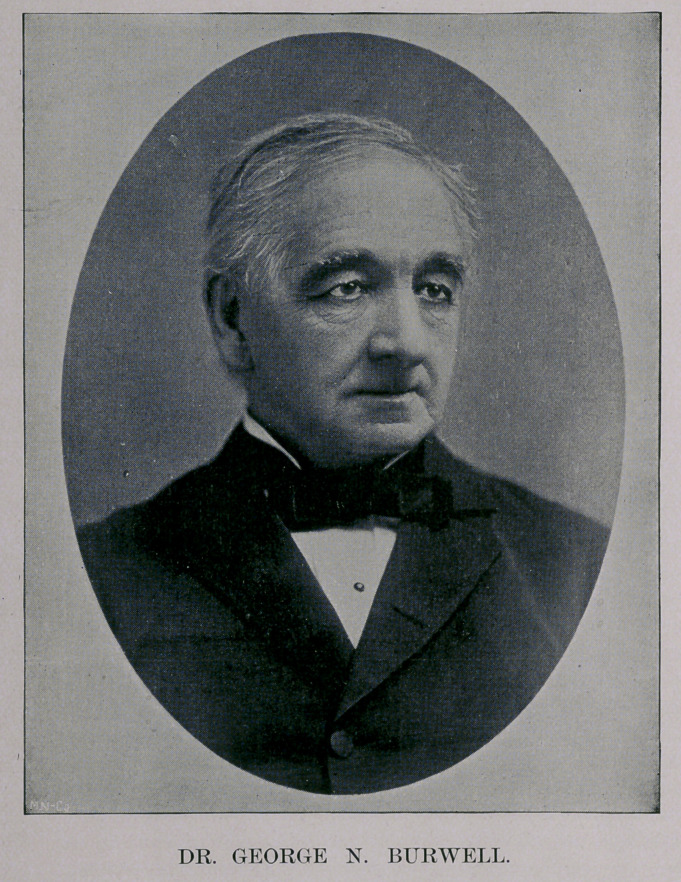# Dr. George N. Burwell

**Published:** 1891-06

**Authors:** 


### Obituary.

Dr. George N. Burwell died at the residence of his sister, Mrs. Wm. H. Glenny, in this city, on Friday evening, May 15, 1891, aged 72 years. For nearly half a century Dr. Burwell had been identified with the medical profession of Buffalo, and had come to be known as one of its strongest and most able members. He was at the time of his death almost the only remaining one of that coterie which has been familiarly spoken of as the old *regime*. Certainly, he was one of the best known physicians in Western New York, and he was particularly endeared to many of the older families in Buffalo. There is scarcely a house within the old city limits that Dr. Burwell has not entered in a professional capacity, either as attending or consulting physician. There is scarcely a family that he has not ministered to some member of, and his name has become endeared to the community by all the attachments which surround the sacred name of family physician.

To those who are familiar with Buffalo during the latter part of the 'forties and early 'fifties, will be recalled the very active part Dr. Burwell took in establishing the Medical Societies on a firm basis for good work. The proceedings of the Buffalo Medical Association during that period present evidences of his regular, conscientious, and active attendance upon the meetings, and the part he bore in the discussions was creditable alike to himself personally as well as to the profession which he loved and served so well. The name of Burwell will always remain a pleasant memory to the medical profession of Buffalo, and it may be said with truth that "take him for all in all we shall not look upon his like again."

His funeral was largely attended from St. Paul's church, on Tuesday, May 19th, and his remains were followed to their last resting place by a large group of mourning friends, while thousands dropped the tears of affection as the cortege wended its solemn way to the place appointed for all the living. He lived a pure and simple life; he died an ideal death. *Requiescat in pace.*

## Figures and Tables

**Figure f1:**